# Association between fibromyalgia and cataract: A database retrospective cohort study

**DOI:** 10.1097/MD.0000000000026447

**Published:** 2021-07-23

**Authors:** Wei-Syun Hu, Cheng-Li Lin, Tung-Sheng Chen

**Affiliations:** a School of Medicine, College of Medicine, China Medical University; b Division of Cardiovascular Medicine, Department of Medicine; c Management Office for Health Data, China Medical University Hospital, Taichung; d School of Life Science, National Taiwan Normal University, Taipei, Taiwan.

**Keywords:** cataract, cohort, fibromyalgia

## Abstract

This study was set to investigate whether fibromyalgia increased cataract risk.

Fibromyalgia patients were the case group and controls were people who never had a history of fibromyalgia. We estimated the hazard ratio of cataract by Cox proportional-hazards model. The adjusted hazard ratios were obtained by controlling variables of age, sex, and comorbidities. Stratification analysis was also performed to ensure the association of fibromyalgia and cataract.

We included 6949 participants in each groups. The incidence rate of cataract in patients with fibromyalgia (108.9 per 1000 person-years) was higher than that of control group (58.9 per 1000 person-years). The risk of cataract in fibromyalgia patients was 2.48 (95% confidence interval = 2.34–2.63) times higher than subjects without fibromyalgia.

Fibromyalgia is associated with higher risk of cataract.

## 1 Introduction

Cataract, a commonly ophthalmic disease resulting in blindness, is increasing recognized as a huge burden in aging society.^[1]^ The pathogenic mechanism of cataract is complicated; oxidative reaction, psychological stress, autonomic dysfunction, immune-mediated causes are possible factors.^[2–5]^ Fibromyalgia, a long time overlooked disorder, has attracted increasing attention recently because of increased incidence.^[6]^ Previous works have proposed for the possible mechanisms of fibromyalgia, such as inflammation response, genic defect, and psychosomatic dysfunction.^[7–11]^ Rather than discussing these 2 distinct disorders, it seems novel and interesting to explore whether the link between these 2 disease are existed. Applying 1:1 matching statistical analysis to limit the difficulty in attempting to adjust for covariates, this retrospective observational study was conducted to describe whether fibromyalgia patients was associated with enhanced risk of cataract using Taiwan National Health Insurance Research Database (NHIRD). In addition, the subgroup analysis of the relationship with age, sex, and comorbidity feature was also sought to analyze.

## 2 Methods

### 2.1 Data source

We analyzed the Longitudinal Health Insurance Database (LHID) to investigate the association between fibromyalgia and cataract.^[12]^ The LHID is a subset of the NHIRD, which contains health information for 99% of the Taiwanese population.^[12,13]^ One million participants were randomly selected into LHID from the NHIRD. The disease code was defined according to the International Classification of Diseases, Ninth Revision, Clinical Modification (*ICD-9-CM*). All identification data were encrypted to avoid ethical concerns. This study was approved by the Institutional Review Board of China Medical University Hospital Research Ethics Committee (CMUH104-REC2-115(CR-6)).

### 2.2 Study population

Fibromyalgia (*ICD-9-CM* 729.1) patients were the case group in the present study. We defined patients with >3 outpatient records of fibromyalgia within three months between 2000 and 2010 as the cases. Controls were people who never had a history of fibromyalgia. Subjects with a history of cataract or aged <20 years were excluded from the study. We matched these 2 groups in the ratio of 1:1 for the distribution of age, sex and medical comorbidities. All participants were followed until the occurrence of cataract, withdrawal from this program, or at the end of the study (31 Dec 2011), whichever came first.

### 2.3 Main outcome and comorbidities

Cataract (*ICD-9-CM* 366) was the primary event of this study. The related comorbidities include hypertension (*ICD-9-CM* 401–405), diabetes (*ICD-9-CM* 250), hyperlipidemia (*ICD-9-CM* 272), chronic obstructive pulmonary disease (*ICD-9-CM* 491, 492, 496), chronic kidney disease (*ICD-9-CM* 585), liver cirrhosis (*ICD-9-CM* 571), asthma (*ICD-9-CM* 493), dementia (*ICD-9-CM* 290, 294.1, 331.0), atrial fibrillation (*ICD-9-CM* 427.3), congestive heart failure (*ICD-9-CM* 428), hyperthyroidism (*ICD-9-CM* 242), sleep disorder (*ICD-9-CM* 307.4, 780.5) and gout (*ICD-9-CM* 274).

### 2.4 Statistical analysis

We applied the *χ*^2^ test and student *t* test to examine the differences of categorical and continuous variables between the case group and the control group. The incidence rate of cataract was calculated in a unit of 1000 person-years. We estimated the hazard ratio of cataract by Cox proportional-hazards model. The adjusted hazard ratios were obtained by controlling variables of age, sex, and comorbidities. Stratification analysis was also performed to ensure the association of fibromyalgia and cataract. Kaplan-Meier method was used to evaluate the cumulative incidence rate of cataract in patients with and without fibromyalgia. We assess the different of the curves by log-rank test.

## 3 Result

We included 6949 participants in each groups (case group: fibromyalgia patients; control group: fibromyalgia-free persons). The case group and the control group tracked an average of 3.63 (± 2.76) years and 6.27 (± 3.43) years, respectively. The baseline demographic characteristics and comorbidities in case cohort and control cohort were presented in Table 1. 56.5% of the subjects were between 50 and 64 years of age, and females accounted for approximately 55% of all participants. The distributions of comorbidities in 2 groups were similar.

**Table 1:** Demographic characteristics and comorbidities in subjects with and without fibromyalgia.

	Fibromyalgia		
Variable	No	Yes	*P*
	N = 6949	N = 6949	
Age, y			.98
≤49	545 (7.84)	539 (7.76)	
50–64	3928 (56.5)	3927 (56.5)	
65+	2476 (35.6)	2483 (35.7)	
Mean ± SD^∗^	61.4 ± 10.5	61.7 ± 9.57	.03
Sex			.97
Female	3819 (55.0)	3817 (54.9)	
Male	3130 (45.0)	3132 (45.1)	
Comorbidity			
Hypertension	3165 (45.6)	3164 (45.5)	.99
Diabetes	797 (11.5)	801 (11.5)	.92
Hyperlipidemia	1758 (25.3)	1765 (25.4)	.89
Chronic obstructive pulmonary disease	884 (12.7)	892 (12.8)	.84
Chronic kidney disease	93 (1.34)	96 (1.38)	.83
Liver cirrhosis	1443 (20.8)	1455 (20.9)	.80
Asthma	515 (7.41)	510 (7.34)	.87
Dementia	52 (0.75)	50 (0.72)	.84
Atrial fibrillation	45 (0.65)	52 (0.75)	.48
Congestive heart failure	251 (3.61)	260 (3.74)	.69
Hyperthyroidism	66 (0.95)	70 (1.01)	.73
Sleep disorder	1225 (17.6)	1219 (17.5)	.89
Gout	712 (10.3)	720 (10.4)	.82

Chi-square test.

∗ *t*-Test. SD = Standard deviation.

**Figure 1:**
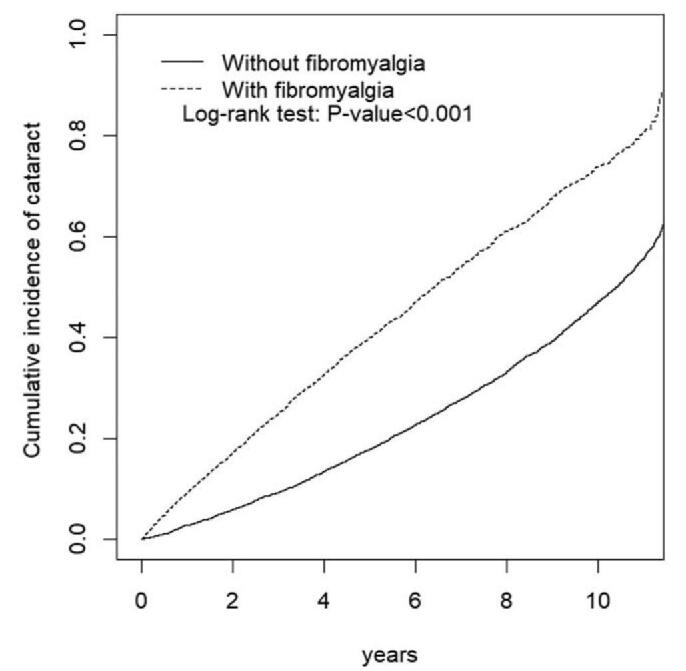
Cumulative incidence curves of cataract for groups with (dashed line) and without (solid line) fibromyalgia.

The cumulative incidence of cataract in case group was obviously higher than that in subjects without fibromyalgia (Fig. 1). The *P* value of log-rank test was <.001. The incidence rate of cataract in patients with fibromyalgia (108.9 per 1000 person-years) was higher than that of control group (58.9 per 1000 person-years) (Table 2). The risk of cataract in fibromyalgia patients was 2.48 (95% confidence interval = 2.34–2.63) times higher than subjects without fibromyalgia. The hazard ratio of cataract for the elderly (≥65 years) compared to people aged ≤49 years was 1.69 (95% confidence interval = 1.49–1.92). Except for atrial fibrillation, patients with comorbidities were more likely to develop cataract than people without comorbidities. The stratification analysis was shown in Table 3. Fibromyalgia increases the risk of cataract in each stratification.

**Table 2:** The incidence and risk factors for cataract.

Variable	Event	PY	Rate^†^	Crude HR (95% CI)	Adjusted HR^§^ (95% CI)
Fibromyalgia					
No	2567	43,591	58.9	1.00	1.00
Yes	2751	25,259	108.9	2.39 (2.25–2.53)^∗∗∗^	2.48 (2.34–2.63)^∗∗∗^
Age, y					
≤49	266	5161	51.5	1.00	1.00
50–64	2208	38,612	57.2	1.08 (0.96–1.23)	0.95 (0.84–1.08)
65+	2844	25,076	113.4	2.08 (1.83–2.36)^∗∗∗^	1.69 (1.49–1.92)^∗∗∗^
Sex					
Female	3060	38,695	79.1	0.98 (0.93–1.03)	
Male	2258	30,155	74.9	1.00	1.00
Comorbidity					
Hypertension					
No	2172	37,829	57.4	1.00	1.00
Yes	3146	31,020	101.4	1.77 (1.67–1.87)^∗∗∗^	1.17 (1.11–1.25)^∗∗∗^
Diabetes					
No	4292	62,045	69.2	1.00	1.00
Yes	1026	6804	150.8	2.32 (2.17–2.49)^∗∗∗^	1.82 (1.70–1.96)^∗∗∗^
Hyperlipidemia					
No	3416	52,317	62.5	1.00	1.00
Yes	1902	16,533	115.1	1.81 (1.71–1.92)^∗∗∗^	1.33 (1.25–1.41)^∗∗∗^
Chronic obstructive pulmonary disease					
No	4342	60,768	71.5	1.00	1.00
Yes	976	8081	120.8	1.77 (1.65–1.89)^∗∗∗^	1.30 (1.20–1.40)^∗∗∗^
Chronic kidney disease					
No	5209	68,126	76.5	1.00	1.00
Yes	109	724	150.6	2.08 (1.72–2.51)^∗∗∗^	1.31 (1.08–1.59)^∗∗∗^
Liver cirrhosis					
No	3921	55,505	70.6	1.00	1.00
Yes	1397	13,345	104.7	1.54 (1.45–1.64)^∗∗∗^	1.21 (1.14–1.29)^∗∗∗^
Asthma					
No	4781	64,137	74.5	1.00	1.00
Yes	537	4712	114.0	1.57 (1.43–1.71)^∗∗∗^	1.17 (1.06–1.28)^∗∗^
Dementia					
No	5261	68,487	76.8	1.00	1.00
Yes	57	362	157.4	2.17 (1.67–2.82)^∗∗∗^	1.48 (1.14–1.92)^∗∗∗^
Atrial fibrillation					
No	5262	68,451	76.9	1.00	1.00
Yes	56	398	140.7	1.91 (1.47–2.48)^∗∗∗^	1.18 (0.90–1.54)
Congestive heart failure					
No	5005	66,745	75.0	1.00	1.00
Yes	313	2104	148.7	2.15 (1.92–2.41)^∗∗∗^	1.26 (1.12–1.42)^∗∗∗^
Hyperthyroidism					
No	5256	68,267	77.0	1.00	1.00
Yes	62	582	106.5	1.45 (1.13–1.87)^∗∗^	1.30 (1.01–1.67)^∗^
Sleep disorder					
No	4063	58,622	69.3	1.00	1.00
Yes	1255	10,227	122.7	1.93 (1.81–2.05)^∗∗∗^	1.64 (1.54–1.76)^∗∗∗^
Gout					
No	4565	62,313	73.3	1.00	1.00
Yes	753	6536	115.2	1.63 (1.51–1.76)^∗∗∗^	1.19 (1.10–1.29)^∗∗∗^

CI = confidence interval, HR = hazard ratio, PY = persona year.

∗ *P* < .05.

∗∗ *P* < .01.

∗∗∗ *P* < .001.

† Rate, incidence rate, per 1000 person-years. Crude HR, relative hazard ratio.

§ Adjusted HR, multivariable analysis including age, sex, and comorbidities of hypertension, diabetes, hyperlipidemia, chronic obstructive pulmonary disease, chronic kidney disease, liver cirrhosis, asthma, dementia, atrial fibrillation, congestive heart failure, hyperthyroidism, sleep disorder, and gout.

**Table 3:** Comparison of incidence and HR of cataract between patients with and without fibromyalgia stratified by age, sex, and comorbidity.

	Fibromyalgia							
	No			Yes				
Variables	Event	PY	Rate^†^	Event	PY	Rate^†^	Crude HR (95% CI)	Adjusted HR^§^ (95% CI)
Age, y								
≤49	113	3253	34.7	153	1908	80.2	2.87 (2.22–3.71)^∗∗∗^	3.03 (2.33–3.94)^∗∗∗^
50–64	1021	24,309	42	1187	14,303	83.0	2.57 (2.35–2.81)^∗∗∗^	2.69 (2.46–2.94)^∗∗∗^
65+	1433	16,029	89.4	1411	9047	156.0	2.24 (2.07–2.42)^∗∗∗^	2.30 (2.12–2.48)^∗∗∗^
Sex								
Female	1467	24,769	59.2	1593	13,925	114.4	2.53 (2.35–2.73)^∗∗∗^	2.62 (2.43–2.83)^∗∗∗^
Male	1100	18,821	58.4	1158	11,333	102.2	2.21 (2.03–2.41)^∗∗∗^	2.33 (2.13–2.54)^∗∗∗^
comorbidity^||^								
No	416	14,158	29.4	469	8577	54.7	2.56 (2.22–2.96)^∗∗∗^	2.57 (2.22–2.96)^∗∗∗^
Yes	2151	29,433	73.1	2282	16,682	136.8	2.43 (2.28–2.58)^∗∗∗^	2.43 (2.28–2.59)^∗∗∗^

CI = confidence interval, HR = hazard ratio, PY = person year.

∗∗∗ *P* < .001.

† Rate, incidence rate, per 1000 person-years Crude HR, relative hazard ratio.

§ Adjusted HR: multivariable analysis including age, sex, and comorbidities of hypertension, diabetes, hyperlipidemia, chronic obstructive pulmonary disease, chronic kidney disease, liver cirrhosis, asthma, dementia, atrial fibrillation, congestive heart failure, hyperthyroidism, sleep disorder, and gout.

|| Comorbidity: Patients with any one of the comorbidities were classified as the comorbidity group: hypertension, diabetes, hyperlipidemia, chronic obstructive pulmonary disease, chronic kidney disease, liver cirrhosis, asthma, dementia, atrial fibrillation, congestive heart failure, hyperthyroidism, sleep disorder, and gout.

## 4 Discussion

This is a retrospective cohort study using a subset data from Taiwan's population-based health insurance registry. The objective was to study an association between fibromyalgia and the incidence of cataract. We selected 6949 patients with and without fibromyalgia, and matched them 1:1 according to age, sex, and comorbidities. Fibromyalgia was associated with an approximately 2.48-fold increase in the hazard of cataract in adjusted analyses, with a stronger association among young age, women, and no comorbidity.

This is an important and very sensitive subject that is obviously not easy to address. The number of observations is significant, and the analysis is performed in a large national database, resulting in the message conveyed by this work clear and strong.

Since this study was retrospective and observational, using a national database, suggesting that we were not able to show the defined clear mechanisms; indeed, only description of association phenomenon instead of providing a causal effect should be acknowledged. Nonetheless, mitochondrial dysfunction, immune system malfunction, and oxidative stress might be possible clues of this link.^[14–18]^ Moreover, genetic defect, autonomic malfunction, and psychologic cause were also possible etiologies for this connection.^[1–11,14–18]^ Therefore, a more comprehensive examination of the potential mechanisms for the powerful association that was observed, as well as some further biologic-pathophysiologic thought about the next steps in research are highly encouraged.

To minimize the impact of baseline differences, we provide strong evidence using a 1:1-matched analysis. We concluded that fibromyalgia is associated with higher risk of incident cataract, and that the risk is higher in the age group ≤49 years of age, and in females and no comorbidity. It shows an increased risk of event, in particular in younger patients and in women. Such an observation is important, and deal with matters we may not pay attention to. In addition, the group with fibromyalgia had a similar density of co-existing comorbidities compared to controls. It is interesting that despite controlling for these factors, there was a higher adjusted HR for cataract among those without comorbidities, further enhancing that the strong association between fibromyalgia and cataract was probably through the comorbidity-independent effect. Based on our observation, such groups deserved further attention so that appropriate preventive strategy and screening program can be adequately instituted.

## 5 Limitations

First, the diagnosis of fibromyalgia is based on records analysis. It is however unclear whether fibromyalgia is the primary diagnosis or not. The etiology of fibromyalgia is also unclear. Moreover, whether all fibromyalgia captured in this registry remained uncertain. Second, record analysis does not provide insights into the patient's background. Finally, the retrospective study based on a coded database has a lot of boundaries and should be acknowledged.

## 6 Conclusions

Association between fibromyalgia and cataract was found.

## Author contributions

Wei-Syun Hu - study concept and design, acquisition of data, analysis and interpretation, drafting of manuscript, critical revision of the manuscript for important intellectual content and study supervision.

Cheng-Li Lin- acquisition of data, analysis and interpretation.

Tung-Sheng Chen- acquisition of data, analysis and interpretation.
